# Latest developments in the use of e-cigarettes by people with schizophrenia spectrum disorders who smoke: a scoping review

**DOI:** 10.3389/fpsyt.2026.1811946

**Published:** 2026-07-21

**Authors:** Pasquale Caponnetto, Sarah Pratt, Carlo Maria Bellanca, Noemi Maria Vitale, Graziella Chiara Prezzavento, Giusy Rita Maria La Rosa, Yusuff Adebayo Adebisi, Ramin Nilforooshan, Isaac John, Abdulnaser Fakhrou, Antonino Petralia, Maria Salvina Signorelli, Uladzimir Pikirenia, Ines Testoni, Roberto Cavallaro, Marta Bosia, Riccardo Polosa

**Affiliations:** 1 Department of Educational Sciences, Section of Psychology, University of Catania, Catania, Italy; 2 Center of Excellence for the Acceleration of Harm Reduction (CoEHAR), University of Catania, Catania, Italy; 3 Azienda Ospedaliero-Universitaria (A.O.U.) Policlinico “G. Rodolico-San Marco”, Psychiatry Unit, University of Catania, Catania, Italy; 4 Department of Psychiatry, Dartmouth-Hitchcock, Geisel School of Medicine at Dartmouth, Hanover, NH, United States; 5 Department of Biomedical and Biotechnological Sciences, Section of Pharmacology, University of Catania, Catania, Italy; 6 Clinical Toxicology Unit, University Hospital of Catania, Catania, Italy; 7 Department of Biomedical Sciences, Section of Neuroscience and Clinical Pharmacology, University of Cagliari, Cagliari, Italy; 8 Department of Clinical and Experimental Medicine, University of Catania, Catania, Italy; 9 College of Social Sciences, University of Glasgow, Glasgow, United Kingdom; 10 Department of Psychiatry, University of Surrey, Guildford, United Kingdom; 11 Royal Holloway, University of London, London, United Kingdom; 12 Department of Psychological Sciences, College of Education University, Doha, Qatar; 13 Department of Clinical and Experimental Medicine, Psychiatry Unit, University of Catania, Catania, Italy; 14 Psychogeriatric Department, Psychiatric Hospital of Frombork, Frombork, Poland; 15 Department of Philosophy, Sociology, Education and Applied Psychology (FISPPA), University of Padua, Padua, Italy; 16 Department of Clinical Neurosciences, Istituto di Ricovero e Cura a Carattere scientifico (IRCCS) San Raffaele Scientific Institute, Milan, Italy

**Keywords:** e-cigarettes, review, schizophrenia spectrum disorders, smoking, vaping

## Abstract

**Background:**

Tobacco use is significantly more prevalent among individuals with schizophrenia spectrum disorders (SSD) compared to the general population, contributing to elevated rates of premature mortality from smoking-related diseases. Despite a decline in smoking prevalence in the general population, individuals with SSD continue to smoke at persistently high rates, driven by biological, psychological, and social factors. Standard smoking cessation approaches yield markedly poorer outcomes in this group compared to non-psychiatric populations. This scoping review aimed to map the emerging evidence on the use of e-cigarettes among individuals with SSD or serious mental illness (SMI).

**Methods:**

This scoping review was conducted in accordance with the Population–Concept–Context (PCC) framework and reported following the PRISMA extension for Scoping Reviews (PRISMA-ScR) guidelines. The review focused on studies published between January 2020 and February 2026, searched on PubMed and EMBASE, providing an up-to-date synthesis of emerging evidence on e-cigarette use in this vulnerable population.

**Results:**

Three studies reported across four publications were included (total N = 323); one research group (Pratt et al.) contributed two separate publications reporting distinct outcomes from the same study cohort. Findings suggest that e-cigarette-based interventions are feasible and acceptable in individuals with SSD and SMI, with preliminary evidence of smoking reduction and decreased exposure to tobacco-related carcinogens. However, sustained harm reduction appeared dependent on a combined approach considering that device provision was empowered in its efficacy to sustain harm reduction over time when integrated with behavioral support.

**Conclusions:**

The available evidence, while preliminary and limited by the small number of included studies, should be interpreted with caution regarding generalizability to patients with Schizophrenia Spectrum Disorders exclusively, as three of the four included publications recruited participants with broader SMI diagnoses of which SSD represents only a subset. Further research, including larger adequately powered trials with standardized outcome measures and longer follow-up, is needed to establish the generalizability and long-term impact of these interventions.

## 1 Introduction

The relationship between e-cigarettes (vaping) and schizophrenia has garnered increasing attention in recent years, particularly as vaping becomes more prevalent among individuals with psychiatric conditions. Among individuals diagnosed with schizophrenia spectrum disorders, the risk of mortality due to smoking-related illnesses is significantly higher compared to non-smokers (1, 2).

Smoking is a leading cause of premature mortality in this population, contributing to tobacco-related cancers, respiratory diseases, and cardiovascular conditions. In addition, smoking in individuals with schizophrenia spectrum disorders (SSD) is linked to depressive symptoms, increased hospitalizations, heightened stress levels, poor treatment outcomes, reduced quality of life, and exacerbation of psychotic symptoms (3).

Despite a decline in smoking prevalence in the general population of developed countries, individuals with SSD continue to smoke at markedly higher rates, estimated at two to four times those without psychiatric disorders (3, 4). They also exhibit greater nicotine dependence (5). Possible explanations include self-medication hypotheses, whereby nicotine transiently improves cognitive function, as well as a complex interplay of illness-related symptoms, patient characteristics, and systemic healthcare barriers (6). While genetic factors have been implicated in both nicotine addiction and schizophrenia progression, psychosocial determinants such as unemployment, social isolation, and the use of smoking to manage stress, boredom, and self-esteem appear equally important (7–9). Attitudes toward smoking cessation within this population present additional challenges. Smoking remains socially accepted within the SSD community, and patients are less likely to be advised to quit by healthcare providers (7, 9–11). Additionally, many clinicians remain concerned that stopping smoking could worsen psychiatric outcomes or deprive patients of one of their few perceived pleasures (4). Together, these systemic and provider-related barriers help explain persistently high smoking rates.

Traditional smoking cessation strategies are substantially less effective in individuals with schizophrenia spectrum disorders than in the general population. Available evidence indicates that commonly prescribed pharmacological treatments, including nicotine replacement therapy (NRT), bupropion, and varenicline, yield only modest cessation outcomes in this group. Among these options, varenicline is often considered the most promising; however, abstinence rates remain markedly lower than those observed in non-psychiatric populations, and ongoing concerns regarding potential neuropsychiatric adverse effects have limited its broader clinical uptake (12). Outcomes associated with NRT have been particularly discouraging, as most studies report long-term abstinence rates below 10% in individuals with schizophrenia, despite its generally favorable safety and tolerability profile (13). Evidence for bupropion points in a similar direction, with meta-analytic findings suggesting minimal benefits for sustained abstinence when compared with placebo (14). Behavioral interventions, such as cognitive-behavioral therapy and motivational interviewing, have shown some effectiveness when delivered intensively, yet their overall impact is constrained by persistent difficulties in engagement and retention, resulting in generally modest effect sizes (15, 16). Within this context, e-cigarettes have attracted increasing attention as a potentially useful smoking cessation tool for individuals with schizophrenia spectrum disorders. By delivering nicotine in a way that more closely replicates the sensory, behavioral, and ritualized aspects of smoking, e-cigarettes may address both the pharmacological and psychological components of nicotine dependence. Elements such as hand-to-mouth movements, visible vapor production, and throat sensation may help satisfy entrenched smoking routines, thereby reducing the appeal of returning to combustible cigarettes. Moreover, the ability to adjust nicotine levels over time allows for more individualized use patterns, which may be particularly important for this population given their typically higher baseline nicotine dependence. In line with this rationale, early empirical evidence suggests that switching from combustible cigarettes to e-cigarettes may contribute to reduced relapse rates and support smoking cessation among individuals with schizophrenia spectrum disorders (17). Beyond schizophrenia spectrum disorders, the relationship between e-cigarette use and mental health has been examined across a broader range of neuropsychiatric conditions. Epidemiological data consistently show that individuals with affective disorders, including major depressive disorder, bipolar disorder, and anxiety-related conditions, use e-cigarettes at substantially higher rates than the general population, with prevalence figures among those with chronic mental illness approximately double those of non-psychiatric controls (18). Longitudinal evidence from a large UK household sample demonstrated that e-cigarette initiation was prospectively associated with worsening general mental health, social dysfunction, and anhedonia over a one-year period (19), while studies in youth and young adults have reported significant associations between e-cigarette use and depression, anxiety, Post-Traumatic Stress Disorder (PTSD) and Attention-Deficit/Hyperactivity Disorder (ADHD) symptoms (20, 21). The directionality of these associations remains debated, with some evidence supporting a self-medication model whereby pre-existing psychiatric vulnerability predisposes individuals to e-cigarette uptake, and other longitudinal data suggesting that e-cigarette initiation itself may worsen certain mental health outcomes (19). Across these diverse diagnostic groups, high rates of combustible tobacco use represent a shared clinical challenge: individuals with bipolar disorder, PTSD, depression, and anxiety disorders also smoke at disproportionately high rates compared to the general population, and similarly exhibit poor outcomes with standard cessation approaches (22). In this broader context, e-cigarettes have been proposed not only in schizophrenia but across the spectrum of serious mental illness as a potentially viable harm reduction strategy for those unable to achieve abstinence through conventional means (23, 24). While earlier reviews have addressed e-cigarette use in mental health populations, most either grouped together diverse psychiatric conditions, relied on limited early evidence, or are now outdated (4, 20). What distinguishes the present scoping review is its focus on the latest developments specific to schizophrenia spectrum disorders, synthesizing new evidence from larger trials, observational studies, and qualitative research. This targeted approach provides a timely update on the potential role of e-cigarettes in addressing one of the most entrenched public health challenges in severe mental illness.

## 2 Materials and methods

This scoping review was conducted in accordance with the Population–Concept–Context (PCC) framework to define the research question and was reported following the PRISMA (Preferred Reporting Items for Systematic Reviews and Meta-Analyses Extension for Scoping Reviews) guidelines (25). Specifically, the Population included individuals with schizophrenia spectrum disorders, the Concept focused on the use of electronic cigarettes, and the Context encompassed all settings, including clinical, community, residential, and outpatient environments. Accordingly, the review addressed the following research question: “*What is the emerging evidence regarding the use of e-cigarettes to quit smoking, reduce smoking or reduce harm among adults with schizophrenia spectrum disorders in clinical and community settings?*”. We chose this well-defined period to capture the latest research conducted in this area.

### 2.1 Search strategy

We conducted an electronic search of PubMed (National Library of Medicine) and EMBASE to identify relevant studies. The search strategy combined terms related to schizophrenia spectrum disorders and vaping: for PubMed, we used the following string: ((“E-Cigarette Vapor”[MeSH Terms] OR “Vaping”[MeSH Terms] OR “electronic nicotine delivery systems”[MeSH Terms] OR “e cig*”[All Fields] OR “Vaping”[All Fields] OR “e-cigarette”[All Fields] OR “vapers”[All Fields] OR “electronic nicotine delivery systems”[All Fields]) AND (“Schizophrenia” [MeSH Terms] OR “Psychotic Disorders”[MeSH Terms] OR (schizo*[Title/Abstract] OR psychotic*[Title/Abstract] OR psychosis[Title/Abstract] OR psychoses[Title/Abstract]))); for EMBASE, we used the following search string: (‘electronic cigarette’/exp OR ‘vaping’/exp OR ‘electronic nicotine delivery system’/exp OR e-cig*:ti,ab OR vaping:ti,ab OR vapers:ti,ab) AND (‘schizophrenia’/exp OR ‘psychotic disorder’/exp OR schizo*:ti,ab OR psychotic*:ti,ab OR psychosis:ti,ab). Furthermore, we performed backward citation searching by manually screening the reference lists of all included studies. Searches were limited to publications between January 2020 and February 2026, with no language or geographical restrictions.

### 2.2 Selection criteria

Studies were included if they met the following conditions:

- Original clinical or epidemiological research involving quantitative data collected from patients, including: prospective cohort studies, randomized or non-randomized clinical trials, pre–post or longitudinal intervention studies, prospective pilot or feasibility studies, mixed-methods studies only if they included a quantitative component. Case series or case reports were eligible only if they presented relevant quantitative data.
- Adults diagnosed with schizophrenia, schizoaffective disorder, or other SSD.
- Use of electronic cigarettes as a method for achieving smoking cessation or smoking reduction.
- Studies reporting quantitative outcomes related to smoking or health, including: smoking behavior (e.g., cigarettes per day, quit rates), biomarkers (e.g., Carbon Monoxide (CO), 4-(methylnitrosamino)-1-(3-pyridyl)-1-butanol (NNAL)), e-cigarette use patterns, measures of nicotine dependence or craving, psychiatric symptoms, adverse events or tolerability. Qualitative outcomes were accepted only within mixed-methods studies that also included quantitative results.

Studies were excluded if they:

- Non-eligible study design, *in vitro* or animal studies; protocols, trial registrations, conference abstracts, posters, or non–peer-reviewed reports, purely qualitative studies without quantitative data, cross-sectional studies (excluded due to lack of prospective data); editorials, commentaries, letters, or reviews (although reviews were screened to identify potentially missing primary studies).
- Studies including participants with schizophrenia spectrum disorders only as part of a broader SMI group without extractable SSD-specific data; focusing exclusively on prodromal psychosis, ultra-high-risk populations, or first-degree relatives without a diagnosed SSD; involving inpatients in acute intoxication/withdrawal or forensic psychiatric populations, unless SSD-specific data were separated.
- Studies evaluating products other than nicotine e-cigarettes, such as HTPs, nicotine pouches, or non-nicotine vaporizers; examining dual use patterns without assessing outcomes related to smoking reduction, cessation, or harm reduction; assessing attitudes or perceptions toward e-cigarettes without measuring behavioral or clinical outcomes.
- Lack of relevant quantitative outcomes related to tobacco use, e-cigarette use, biomarkers, or clinical effects.

### 2.3 Study selection

Four reviewers independently screened all records identified through database searches. The screening occurred in two stages: Title and abstract screening, to identify potentially relevant studies according to the predefined inclusion criteria; Full-text screening, conducted independently by both reviewers to confirm final eligibility. Discrepancies were resolved through discussion or consultation with a third reviewer.

### 2.4 Data extraction

A standardized extraction sheet was used to collect the following information from each included study: Study design and setting; Sample size and diagnostic characteristics (schizophrenia, schizoaffective disorder, other SSD); Type and specifications of the e-cigarette device; Nicotine concentration and usage protocol; Outcomes related to smoking behavior (cigarettes/day, reduction, quit rates); Biomarkers (exhaled CO, NNAL, cotinine); Measures of nicotine dependence and craving; Psychiatric outcomes (PANSS, BPRS, side effects); Adverse events and tolerability; Duration of follow-up; Type of behavioral support, if any. Data extraction was performed independently by two reviewers; inconsistencies were resolved by consensus.

### 2.5 Data synthesis

Quantitative pooling was not possible due to variations in follow-up duration, device type, comparator conditions, and measurement methods. Given the heterogeneity of study designs, populations, and outcomes, a narrative synthesis approach was used. Findings were grouped according to key outcome domains: Feasibility and acceptability; Safety and psychiatric stability; Smoking reduction and cessation outcomes; Biomarker-based harm reduction; Device characteristics and intervention components.

## 3 Results

### 3.1 Studies

**Figure 1:**
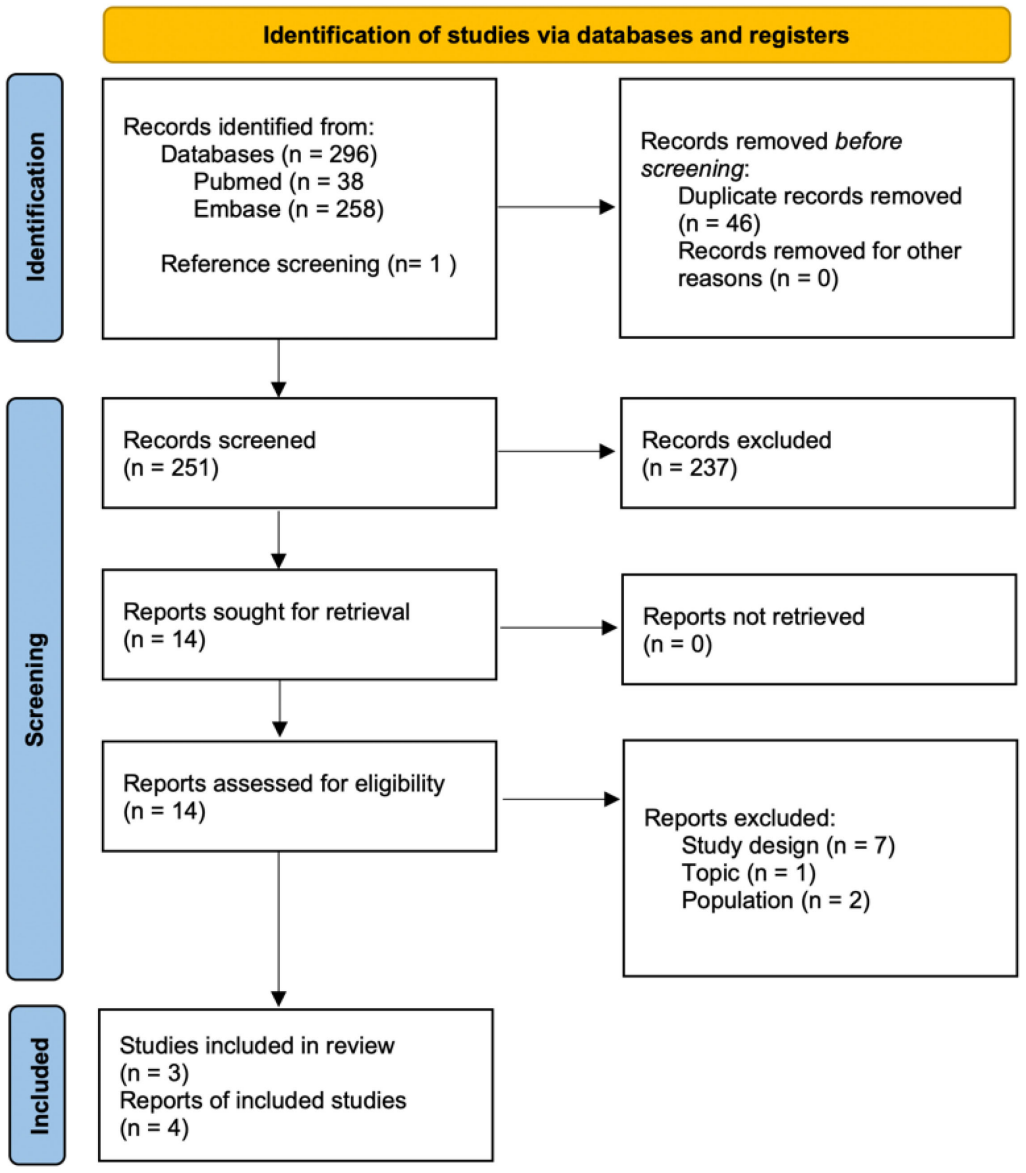
PRISMA flow diagram.

The search process is illustrated in **Figure 1**, according to PRISMA 2020. The initial search yielded 297 records. After removing 46 duplicates, the remaining articles were screened to identify potentially eligible studies. A list of all studies excluded after full-text screening, including reasons for exclusion, has been provided in **Supplementary Table 1**. Finally, 3 studies reported across 4 publications were included for the synthesis; one research group (Pratt et al.) contributed two separate publications reporting distinct outcomes from the same study cohort. The main characteristics of the included studies are reported in **Table 1**.

**Table 1:** General characteristics of the included studies.

Author & year	Title	Link	Study type	Sample size (n)	Diagnostic composition of sample	Device specifications	Nicotine concentration	Impact of e-cigarettes on schizophrenia	Behavioural support
Caponnetto, P., DiPiazza, J., Kim, J., Maglia, M., & Polosa, R., 2021 (17)	A Single-Arm, Open-Label, Pilot, and Feasibility Study of a High Nicotine Strength E-Cigarette Intervention for Smoking Cessation or Reduction for People With Schizophrenia Spectrum Disorders Who Smoke Cigarettes	https://pubmed.ncbi.nlm.nih.gov/33723598/	Single-arm (non-randomized) pilot study	40	SSD only (schizophrenia, schizoaffective disorder)	JUUL closed-system pod device	5%	High nicotine e-cigarettes led to reduced cigarette consumption and were well-tolerated, suggesting a viable cessation tool for schizophrenia patients.	Providing information on e-cigarettes as a potentially less harmful alternative to traditional cigarettes, offering guidance on behavioural substitution, encouraging self-monitoring of cigarette consumption via study diaries, and providing the option to contact the researchers by telephone for technical and medical assistance. No formal smoking cessation counselling was provided, nor were any structured behavioural interventions implemented.
Pratt, S.I. et al., 2022 (26)	E-Cigarette Provision to Promote Switching in Cigarette Smokers With Serious Mental Illness—A Randomized Trial	https://academic.oup.com/ntr/article/24/9/1405/6562456	RCT	68	Broader SMI (schizophrenia, schizoaffective disorder, bipolar disorder, major depressive disorder with psychotic features); no SSD-specific subgroup analysis reported	NJOY Daily e-cigarette	Disposable e-cigarette provided up to 300 puffs, roughly the equivalent of 20 cigarettes	Article presents the results of a randomized trial assessing the impact of e-cigarette provision on smoking behavior among individuals with serious mental illness (SMI), including schizophrenia and bipolar disorder. Key findings indicate that providing e-cigarettes led to significant reductions in daily cigarette consumption (up to 50%) and breath carbon monoxide (CO) levels during an 8-week intervention period. Importantly, 19–22% of participants fully switched from cigarettes to e-cigarettes during this time.	Minimal-contact design, in which the Study Coordinator provided only brief safety instructions and device instructions with no additional coaching.
Kale, D. et al., 2025 (27)	Providing an e-cigarette starter kit for smoking cessation and reduction as adjunct to usual care to smokers with a mental health condition: findings from the ESCAPE feasibility study	https://doi.org/10.1186/s12888-024-06387-7	RCT	43	SSD (not isolated as a distinct subgroup from other mental disorder)	Aspire PocketX: third-generation open-system device	Participants were offered a choice of three nicotine strengths: 0.6%, 1.0%, or 1.8%	E-cigarette provision was acceptable and showed promising harm-reduction outcomes: at 1-month, 61.9% of the intervention group reported ≥ 50% reduction in cigarette consumption (vs 13.6% control), 9.5% had CO-validated abstinence (vs 0% control). No major negative mental-health effects reported; the intervention was well tolerated.	E-cigarette starter kit alongside VBA, a brief product demonstration, and written information, delivered as an adjunct to usual care.
Pratt, S.I. et al., 2025 (28)	Carcinogen reduction in a randomized controlled study comparing e-cigarette provision to assessment only among people with serious mental illness who smoke.	https://doi.org/10.1016/j.drugalcdep.2025.112740	RCT	240	Broader SMI (schizophrenia, schizoaffective disorder, bipolar disorder, major depressive disorder with psychotic features); no SSD-specific subgroup analysis reported	NJOY Daily e-cigarette	Disposable e-cigarette provided up to 300 puffs, roughly the equivalent of 20 cigarettes	E-cigarette provision led to a significant short-term reduction in NNAL levels at 4 weeks compared to assessment-only, indicating reduced exposure to tobacco-related carcinogens. However, this effect attenuated by 8 weeks, suggesting that e-cigarette provision alone may not sustain harm reduction over time without additional behavioral support.	Minimal-contact design, in which the Study Coordinator provided only brief safety instructions and device instructions with no additional coaching.

RCT, randomized controlled trial; SMI, serious mental illness; SSD, schizophrenia spectrum disorders; VBA, Very Brief Advice.

The review included three prospective studies, reported across four publications. These comprised one pilot trial (17; n=40) and three randomized controlled trials (RCTs) (26, n=68; 28, n=240; 27, n=43). The studies evaluated the impact of e-cigarettes on smokers with schizophrenia spectrum disorders or SMI. With regard to diagnostic composition, the study by Caponnetto et al. (17) enrolled exclusively individuals with schizophrenia spectrum disorders, as defined by DSM-5 criteria, including schizophrenia and schizoaffective disorder. In contrast, the two trials by Pratt et al. (26, 28) recruited participants with a broader SMI diagnosis, encompassing schizophrenia, schizoaffective disorder, bipolar disorder, and major depressive disorder with psychotic features, without reporting outcomes stratified by specific diagnosis. Similarly, the study by Kale and Beard (27) included participants with SSD and other mental disorders, representing an even more heterogeneous population. As a result, only one of the four included publications focused exclusively on SSD, while the remaining three relied on broader diagnostic categories of which SSD constitutes a subset. This diagnostic heterogeneity is an important consideration when interpreting and generalizing the findings of this review, as discussed in the Limitations section. Three studies were conducted in the United States and one in Italy (17, 26–28). The total sample size across unique study cohorts was 323 participants (54.8% male). Study durations ranged from 4 weeks to 26 weeks. Outcome measures included biochemically verified abstinence, cigarette consumption reduction, biomarkers of toxicant exposure [urinary 4-(methylnitrosamino)-1-(3-pyridyl)-1-butanol (NNAL), exhaled carbon monoxide (CO)], psychiatric symptomatology scales, and feasibility/acceptability assessments. The findings provide preliminary evidence of positive effects across multiple domains, including high feasibility and acceptability of e-cigarette-based interventions, good safety and psychiatric tolerability, measurable reductions in cigarette consumption, and preliminary indicators of harm reduction such as decreases in toxicant exposure.

### 3.2 Feasibility and acceptability

The pilot and feasibility trials uniformly indicated that e-cigarette-based interventions are acceptable and feasible for individuals with serious mental illness, including those with SSD. The protocol developed by Caponnetto, which used high-nicotine concentration devices, was validated as both feasible and well-tolerated (17). Similarly, Kale demonstrated strong uptake and retention rates when e-cigarette starter kits were provided as an adjunct to usual care, confirming the practicality of implementing such interventions in larger RCTs (27).

### 3.3 Smoking reduction and abstinence outcomes

In a more recent single-arm prospective trial employing high-nicotine electronic nicotine delivery systems, Caponnetto reported substantial reductions in combustible cigarette use (17). At 12-week follow-up, 87.5% of participants achieved at least a 50% reduction in cigarette consumption, while 40% attained biochemically verified abstinence. Overall, these findings indicate that e-cigarettes may support both meaningful smoking reduction and short-term abstinence among individuals with schizophrenia spectrum disorders or severe mental illness.

### 3.4 Biomarker-based harm reduction

Two RCTs provided biochemical evidence relevant to harm reduction. Pratt et al. (28) observed a significantly greater reduction in urinary NNAL among participants assigned to e-cigarettes compared with controls, as indicated by a significant Group × Time interaction (*F* = 3.68, *p* <.026). This reduction was most pronounced at 4 weeks (*p* <.02) but diminished by week 8 (*p* <.07), a pattern directly attributable to widespread continued dual use. Despite active e-cigarette provision, approximately 80% of participants in the intervention group continued to use combustible cigarettes at weeks 2 and 8, compared to 100% of the assessment-only group; by week 26, following cessation of device provision, dual use in the intervention group rose further to 91.7%. Consistent with this, one-way ANOVAs confirmed that participants with CO ≥ 10 ppm, indicative of ongoing combustible cigarette use, had significantly higher NNAL levels than those with lower CO values at both 4 and 8 weeks (*F* = 10.85, *p* <.001 and *F* = 9.04, *p* <.0001, respectively). In the companion trial, Pratt et al. (26) found no significant between-group differences in exhaled CO at weeks 13 or 26, despite the e-cigarette group having reduced cigarette consumption by 59% versus only 6% in the assessment-only group during the provision phase. Overall, these biomarker outcomes point to partial but clinically meaningful harm reduction, with the strongest effects observed when e-cigarettes replace, rather than supplement, combustible cigarettes. These findings should nonetheless be interpreted with caution in the context of SSD specifically, as both Pratt trials recruited participants with broader SMI diagnoses; in the absence of diagnosis-stratified analyses, it is not possible to determine whether the observed reductions in NNAL and exhaled CO are generalizable to individuals with SSD or are substantially influenced by participants with other SMI diagnoses such as bipolar disorder or major depressive disorder with psychotic features.

## 4 Discussion

This scoping review identified three studies, reported across four publications examining the use of e-cigarettes among individuals with schizophrenia spectrum disorders and SMI. The included evidence, comprising one pilot trial and three RCTs, provides preliminary but converging support for e-cigarettes as a feasible, acceptable, and potentially effective harm reduction tool in this high-risk population. Across all included studies, the findings show preliminary but promising evidence, including good feasibility and acceptability, good safety and psychiatric stability, meaningful smoking reduction, and measurable reductions in carcinogen exposure. At the same time, the attenuation of biomarker improvements over time and limited changes in CO underscore the importance of combining e-cigarettes with structured behavioral support to maximize complete substitution and sustain harm reduction benefits. These findings are particularly significant given the substantial health disparities associated with tobacco use among individuals with SMI and the historical challenges in implementing effective smoking cessation interventions in this context.

The demonstration of feasibility and acceptability across all the included studies represents a critical foundational achievement. Individuals with schizophrenia and other SMI have notably high rates of nicotine dependence, often related to self-medication hypotheses and neurobiological factors influencing reward processing and cognitive function (4). Traditional smoking cessation methods, including NRT and pharmacological interventions, have demonstrated limited effectiveness in this population due to low adherence rates, concerns about medication side effects, and the reinforcing behavioral aspects of smoking (13, 16). The pilot trial by Caponnetto validated the feasibility and tolerability of high-nicotine concentration e-cigarettes, while Kale demonstrated strong uptake and retention rates when e-cigarette starter kits were provided as an adjunct to usual care (17, 27). The consistent finding that, in the short-term trials included, e-cigarettes did not worsen psychotic symptomatology, or that no acute exacerbation of symptoms was observed, and were associated with minimal dropout rates due to adverse effects establishes a crucial safety profile that has often been questioned in clinical practice (17, 27). E-cigarettes offer an alternative that not only delivers nicotine in a potentially less harmful manner but also mimics the behavioral rituals associated with smoking, addressing both the physiological and psychological components of addiction.

The smoking reduction outcomes documented in the included studies demonstrate meaningful clinical effects. The trial by Caponnetto reported that 87.5% of participants achieved at least 50% reduction in cigarettes per day, with 40% achieving biochemically verified abstinence at 12 weeks (17). These effect sizes compare favorably with historical outcomes using traditional cessation methods in SMI populations and suggest that interventions involving e-cigarettes warrant serious consideration as part of comprehensive tobacco treatment strategies (10, 13). The variability in outcomes across studies likely reflects differences in intervention intensity, device characteristics, nicotine concentrations, and the degree of behavioral support provided alongside device provision. The use of higher nicotine concentrations in more recent trials suggests that device specifications and nicotine delivery efficiency may be important determinants of intervention success (17). These findings align with broader observational evidence from McGirr, who found that 85% of 270 smokers with mental health and substance use disorders chose to use e-cigarettes, and those participating in structured eight-week switching programs achieved abstinence rates of 12-24% and smoking reduction rates of 29-66% after six months, highlighting the importance of sustained behavioral support (29). The biomarker evidence from the RCTs by Pratt provides direct toxicological support for the harm reduction potential of e-cigarettes while simultaneously revealing important limitations of device provision alone. Pratt demonstrated a significantly greater reduction in urinary NNAL levels, a biomarker of exposure to tobacco-specific nitrosamines, in the e-cigarettes group compared to controls, with the strongest effect observed at 4 weeks (28). This finding offers tangible evidence that e-cigarette use can meaningfully decrease exposure to carcinogenic compounds compared to continued combustible cigarette smoking. However, the attenuation of this effect at 8 weeks, alongside the absence of significant differences in exhaled carbon monoxide levels at 26 weeks in the companion trial, indicates that simply providing devices is insufficient to ensure sustained harm reduction or complete substitution of combustible cigarettes (26). The observed patterns suggest that many participants may have continued dual use of both e-cigarettes and combustible cigarettes, or that adherence to e-cigarette use decreased over time in the absence of adequate behavioral support. The contrast between outcomes with and without structured behavioral support, as evidenced by McGirr, underscores the importance of coupling e-cigarette provision with comprehensive counselling and ongoing monitoring to maximize substitution of combustible cigarettes and sustain harm reduction benefits (29).

Beyond these primary outcomes, recent reviews highlight important clinical complexities of e-cigarette use among people with schizophrenia, particularly those treated with clozapine. Harry showed that switching from smoking to vaping can lead to substantial increases in plasma clozapine levels because the removal of combustion products reduces CYP1A2 induction (30). Several case reports describe instances of clozapine toxicity following transitions from cigarettes to e-cigarettes, with symptoms such as sedation, seizures, and worsening psychosis (31, 32). Data suggests that changes in smoking behavior can have clinically relevant pharmacokinetic consequences in individuals treated with clozapine, including alterations in plasma clozapine levels, as reflected by changes in the clozapine to norclozapine ratio, with potential downstream effects on tolerability, cardiometabolic risk, and cognitive functioning (33). In light of clozapine’s unique therapeutic profile and its central role in the management of treatment-resistant schizophrenia, the importance of careful clinical monitoring during any modification of smoking habits has been strongly highlighted in the literature (34). Consequently, although e-cigarettes may represent a viable harm reduction option, their use in patients receiving clozapine should be accompanied by individualized pharmacological monitoring to mitigate the risk of adverse outcomes.

Beyond this specific pharmacokinetic consideration, the findings of this review must be situated within the wider literature on e-cigarette use across neuropsychiatric conditions beyond schizophrenia. From a clinical implementation standpoint, the variation in behavioral support across included studies provides useful benchmarks for practitioners. Caponnetto et al. (17) offered a minimal behavioral support protocol consisting of verbal and written psychoeducation about e-cigarettes as a potentially less harmful substitute, behavior substitution instructions (replacing cigarettes with e-cigarettes), and self-monitoring of daily cigarette consumption via structured study diaries. Researchers were also contactable by telephone between visits for technical and medical assistance. Participants attended five in-person clinic visits at baseline, weeks 4, 8, 12, and 24, with device supplies replenished at baseline, week 4, and week 8. No formal smoking cessation counselling was provided. The Pratt trials (26, 28) adopted an even more minimal approach, deliberately limiting behavioral input to a single brief session covering device safety information and an instruction to try to replace all cigarettes with e-cigarettes, with no further coaching or counselling. Devices were replenished at biweekly study visits occurring at weeks 0, 2, 4, 6, and 8, with follow-up assessments at weeks 13 and 26. This design was intentional, aimed at isolating the effect of device provision alone from any behavioral component. In contrast, the ESCAPE feasibility study (27) integrated Very Brief Advice (VBA), a structured, evidence-based cessation support technique delivered opportunistically within routine clinical appointments, alongside practical device demonstration and written guidance. This model required no dedicated appointment time, as support was embedded within existing consultations, representing a low-burden, scalable approach suitable for community mental health settings. Taken together, these designs span a useful continuum for clinical practice. The Pratt evidence demonstrates that even minimal-contact device provision can initiate meaningful smoking reduction; however, the attenuation of effects after device supply ended suggests that this approach alone is insufficient to sustain switching. The ESCAPE model indicates that brief, structured support integrated within routine appointments, including device demonstration, harm reduction messaging, and written instructions, may provide sufficient scaffolding to initiate and maintain substitution without requiring dedicated cessation clinics or specialist referral. For clinicians considering implementation, the available evidence suggests that a brief introductory session including device familiarization, clear substitution instructions, and at least biweekly follow-up during the active switching phase, combined with more intensive support thereafter to consolidate switching and prevent relapse to combustible cigarettes, represents a clinically feasible and evidence-informed approach.

Research consistently documents elevated rates of e-cigarette use among individuals with affective disorders, anxiety disorders, PTSD, and ADHD, populations that share with SSD the burden of high smoking prevalence and limited effectiveness of first-line cessation treatments (18, 22). In the general population, longitudinal evidence indicates that e-cigarette initiation is prospectively associated with worsening mental health outcomes including social dysfunction, anhedonia, and loss of confidence (19), and population-based studies confirm associations between e-cigarette use and depression, anxiety, and psychological distress more broadly (20, 21). These findings are clinically important and should not be dismissed. However, as with schizophrenia, the risk-benefit calculus shifts fundamentally when the comparator is continued combustible cigarette smoking rather than non-use. A secondary analysis of a large RCT demonstrated that adding e-cigarettes to standard counselling significantly increased abstinence rates in participants using psychotropic medications, a proxy for psychiatric comorbidity, suggesting that the harm reduction potential of e-cigarettes extends beyond SSD to the broader population of psychiatric smokers (24). This convergent evidence supports a population-sensitive clinical approach: while caution is warranted in never-smokers and in the general population, e-cigarettes may represent a pragmatic and potentially life-saving option across the spectrum of serious mental illness in individuals who smoke and are unwilling or unable to quit through conventional means. From a broader public health perspective, the need for effective harm reduction strategies in this population is further underscored by epidemiological data on smoking-related morbidity and mortality. Chronic obstructive pulmonary disease has been identified as the leading cause of premature death among individuals with schizophrenia in the United States, with schizophrenia itself emerging as an independent risk factor for COPD even after accounting for smoking history (35). The current findings reinforce the urgency of developing targeted cessation and harm reduction approaches for this high-risk group. In this regard, growing attention has been directed toward the potential of e-cigarettes to reduce disparities in tobacco use disorder among individuals with mental health and substance use conditions. While uncertainties regarding long-term safety remain, the available evidence suggests that the potential harm reduction benefits justify continued investigation, particularly with careful consideration of vulnerable populations (23).

Epidemiological evidence highlights patterns of e-cigarette use that are particularly informative for understanding both the opportunities and potential risks associated with these products in populations with serious mental illness. Large-scale observational data show that individuals with mental health conditions are more than twice as likely to use e-cigarettes compared to those without such conditions, with use often motivated by perceived benefits related to relaxation and stress management (18). Complementary findings suggest that continued use among smokers with serious mental illness is influenced by multiple factors, including the perception of reduced harm relative to combustible cigarettes, positive subjective effects, and greater ease of use (36). Economic considerations also appear to play a role, as rising cigarette prices have been associated with increased interest in e-cigarettes, supporting their potential function as more affordable alternatives within vulnerable populations (37). Alongside these quantitative data, qualitative studies offer important insights into lived experiences with smoking cessation and harm reduction. Participants in qualitative interviews frequently described e-cigarettes as appealing due to their flexibility, lower perceived stigma, and similarity to conventional smoking practices (38). Further qualitative evidence reports that dissatisfaction with traditional pharmacological and behavioral interventions, coupled with inconsistent guidance from healthcare providers, may lead some individuals with schizophrenia to explore non-combustible alternatives. In this context, e-cigarettes and related products were often perceived as cleaner and more socially acceptable than combustible cigarettes (39). Together, these qualitative accounts are consistent with the feasibility and acceptability outcomes observed in the quantitative studies included in this review, suggesting that the behavioral and ritualistic dimensions of e-cigarette use may meaningfully contribute to their appeal and potential effectiveness.

Social and family perspectives further contextualize these findings. Evidence from caregivers and close social networks reveals that e-cigarettes are often viewed as a less harmful option for individuals with schizophrenia who smoke daily, with many family members expressing support for their inclusion within harm reduction strategies (9). The observed dynamics underscore the relevance of integrating social support processes into smoking cessation and harm reduction interventions that involve e-cigarettes.

The growing body of evidence supporting e-cigarettes as a harm reduction tool raises important questions for policy and clinical practice in individuals with schizophrenia spectrum disorders. International positions vary substantially, reflecting deeper differences in regulatory philosophy and the weight assigned to harm reduction as a public health principle. The UK National Health Service endorses e-cigarettes as an effective cessation aid, supported by evidence of increased quit rates compared to nicotine replacement therapy (40). Australia’s RANZCP supports e-cigarettes as a second-line option for people with severe mental illness when first-line treatments have failed, acknowledging that the risk-benefit calculus shifts fundamentally in populations where conventional cessation approaches yield persistently poor outcomes (41). In contrast, the US Food and Drug Administration has not approved e-cigarettes as cessation therapies, and has not granted marketing authorization specifically for use as cessation devices, although it has authorized certain e-cigarette products for sale through the Premarket Tobacco Product Application (PMTA) pathway. For clinicians in restrictive jurisdictions, this landscape creates a genuine ethical tension. An ethically defensible approach involves: transparent, individualized shared decision-making presenting e-cigarettes as an unapproved but increasingly studied option; conditional use after failure of first-line treatments, with structured follow-up and pharmacokinetic monitoring, particularly for patients receiving clozapine; and careful documentation within a harm reduction framework that prioritizes the patient’s overall health trajectory.

Some limitations need to be mentioned. Only three unique studies involving 323 participants were identified, restricting the strength and generalizability of the conclusions. Furthermore, the aggregate sample of 323 participants across only three studies is substantially underpowered to detect rare but clinically significant adverse events, such as seizures or severe cardiac events, or to identify subtle changes in psychiatric symptomatology. This represents a primary limitation. All studies were conducted in high-income countries (United States and Italy), both characterized by well-developed mental health infrastructure, established regulatory frameworks governing e-cigarette products, and relatively stable access to healthcare services. This geographic concentration substantially limits the generalizability of findings to low- and middle-income countries (LMICs), where the clinical and contextual conditions surrounding tobacco use in schizophrenia spectrum disorders may differ markedly. Smoking prevalence among individuals with schizophrenia in LMICs is influenced by distinct socioeconomic determinants, including poverty, limited access to pharmacological cessation treatments, and weaker tobacco control policies, which may alter both the baseline risk profile and the relative benefit of harm reduction strategies such as e-cigarettes. Furthermore, the specific devices evaluated in the included studies may not be accessible or affordable in LMIC settings, rendering direct translation of these findings problematic. The near-total absence of data from LMICs represents a critical gap in the literature, particularly given the disproportionate burden of both tobacco-related morbidity and severe mental illness in these settings. Future research should therefore prioritize the inclusion of participants from diverse geographical and economic contexts to establish whether e-cigarette-based harm reduction strategies are feasible, acceptable, and effective across the full range of settings in which individuals with schizophrenia spectrum disorders receive care.

A further limitation concerns the diagnostic composition of the included studies. Only one of the four included publications enrolled exclusively individuals with schizophrenia spectrum disorders (17), while the remaining three recruited participants with broader SMI diagnoses without reporting outcomes stratified by specific diagnosis. As a result, findings may not be fully generalizable to patients with Schizophrenia Spectrum Disorders exclusively, Consequently, findings cannot be directly generalized to patients with Schizophrenia Spectrum Disorders without important caveats, as the observed effects on smoking reduction, biomarkers, and feasibility may be substantially driven by participants with other SMI diagnoses such as bipolar disorder or major depressive disorder with psychotic features. Diagnosis-stratified analyses or studies enrolling exclusively SSD populations are needed before definitive conclusions can be drawn for this specific group. Future studies should prioritize diagnosis-stratified analyses or enroll exclusively SSD populations to allow more definitive conclusions.

As Szerman underlined, a dual-disorder perspective is needed in addressing tobacco use among individuals with mental health conditions, advocating for the integration of e-cigarettes into comprehensive treatment plans that also include behavioral counseling, pharmacotherapy, and careful monitoring of both psychiatric symptoms and medication levels (42). Mendelsohn highlights that e-cigarettes can represent a safe and effective option for patients with severe mental illness who are unable to quit smoking using standard treatments, but this should be understood within the context of individualized, multidisciplinary care rather than as a simple intervention that can be implemented without adequate support and monitoring (43).

The growing body of research continued to support the feasibility, acceptability, and effectiveness of e-cigarettes and other alternative nicotine delivery systems in reducing cigarette consumption and promoting cessation (29). These strategies offer a potentially lower-risk alternative while addressing both the behavioral and neurobiological aspects of nicotine addiction. While further research is needed to fully understand the long-term implications of e-cigarette use, current evidence suggests that incorporating e-cigarettes into a harm reduction framework could play a crucial role in reducing smoking-related health disparities among individuals with severe mental illness. Future studies should focus on optimizing the role of e-cigarettes within multidisciplinary treatment models, taking into account patient preferences, evolving clinical guidelines, and regulatory considerations to ensure safe and effective implementation.

## Data Availability

The original contributions presented in the study are included in the article/**Supplementary Material**. Further inquiries can be directed to the corresponding author.

## Supplementary material

Supplementary Table 1 Excluded studies after full-text reading.
