# Supplementary material for: Latest developments in the use of e-cigarettes by people with schizophrenia spectrum disorders who smoke: a scoping review
Source: Front Psychiatry. 2026 Jul 21;17:1811946. doi: 10.3389/fpsyt.2026.1811946 (PMC13433363; doi:10.3389/fpsyt.2026.1811946)
Supplement: Supplementary file 1 [file Table1.docx]

**Supplementary Table 1**. Excluded studies.

| **Nr** | **Author & Year** | **Title** | **Link** | **Reason for exclusion** |
| --- | --- | --- | --- | --- |
| 1. | Taylor, E., et al. (2025) | Tobacco-related toxicant exposure among people with and without experience of psychosis: findings from the US Population Assessment of Tobacco and Health study | <https://doi.org/10.1136/bmjopen-2025-101066> | Cross-sectional |
| 2. | Gardner, M., et al. (2025) | Field-Portable Device for Detection of Controlled and Psychoactive Substances from e-Cigarettes | <https://doi.org/10.1021/acsomega.4c08614> | Focus on another topic |
| 3. | Bennett, M. E. et al. (2024) | Tobacco smoking and nicotine vaping in persons with first episode psychosis | <https://doi.org/10.1016/j.schres.2024.03.020> | No people with schizophrenia spectrum disorders |
| 4. | Do, E. K. et al. (2024) | Past-Year Mental Illness Diagnosis and E-cigarette Use Status Among a College-Aged Sample: Findings From the National College Health Assessment (2017-2019) | <https://doi.org/10.1177/08901171231224864> | Cross-sectional survey |
| 5. | Hyatt, A. S. et al. (2024) | Association between electronic nicotine product use and subsequent first episode psychosis | <https://doi.org/10.1016/j.psychres.2024.115785> | No people with schizophrenia spectrum disorders |
| 6. | Han, B. et al. (2023) | Tobacco Use, Nicotine Dependence, and Cessation Methods in US Adults With Psychosis | <https://doi.org/10.1001/jamanetworkopen.2023.4995> | Cross-sectional |
| 7. | Caponnetto, P., et al. (2023) | Psychological Experience of Smoking Addiction in Family and Friends of Schizophrenic Adults Who Smoke Daily: A Qualitative Study | <https://doi.org/10.3390/healthcare11050644> | Qualitative study |
| 8. | Kertes, J., et al. (2021) | Comparison of Perceptions and Smoking Cessation Experiences Between Smokers With and Without Serious Mental Illness in a Large Health Maintenance Organization | <https://doi.org/10.1080/15504263.2021.1979348> | Cross-sectional |
| 9. | Caponnetto, P., & Polosa, R. (2021). | A qualitative study of the views about smoking, licensed cessation aids and e-cigarettes in people with schizophrenia spectrum disorders | <https://doi.org/10.1080/10550887.2020.1826097> | Qualitative study |
| 10. | Wong, J. A., et al. 2022 | Characteristics of and reasons for electronic cigarette use among adult smokers with schizophrenia/schizoaffective disorder. | <https://doi.org/10.12788/acp.0050> | Longitudinal-descriptive study |
